# Factors Associated With Anxiety and Depression in Persons With Epilepsy (PWE)

**DOI:** 10.7759/cureus.56401

**Published:** 2024-03-18

**Authors:** Spyridon Roussos, Georgia Gerogianni, Georgios Vasilopoulos, Ioannis Koutelekos, Niki Pavlatou, Antonia Kalogianni, Georgia Toulia, Afroditi Zartaloudi, Maria Polikandrioti

**Affiliations:** 1 Department of Nursing, Postgraduate Program "Applied Clinical Nursing", University of West Attica, Athens, GRC; 2 Department of Nursing, University of West Attica, Athens, GRC

**Keywords:** persons with epilepsy, neurological disorder, mental wellbeing, epilepsy, depression, anxiety

## Abstract

Introduction: Epilepsy is a common neurological disease that is associated with increased morbidity and mortality globally. Persons with epilepsy (PWE) experience a heavy emotional burden mainly due to social stigmatization and limited opportunities in life. The purpose of this study was to explore factors associated with anxiety and depression in PWE.

Material and methods: In the present cross-sectional study, we enrolled 100 PWE who visited outpatient settings in a public hospital for scheduled follow-up. Data collection was carried out by the completion of the Hospital Anxiety and Depression Scale (HADS), which included participants’ characteristics. The statistical significance level was p < 0.05.

Results: Of the 100 participants, the majority were women (65%), below 50 years old (62%), and single (40%). In terms of mental comorbidity, 58% and 48% experienced anxiety and depression, respectively. A statistically significant association was observed between anxiety and age (p = 0.002). Furthermore, a statistically significant association was observed between depression and gender (p = 0.044), age (p = 0.001), marital status (p = 0.036), educational level (p = 0.003), job (p = 0.025), residency (p = 0.041), and whether they went out at night (p = 0.009).

Conclusion: Identifying factors associated with anxiety and depression is essential for PWE to receive appropriate support.

## Introduction

Epilepsy is a major neurological disorder that affects approximately 65 million people globally [1] and over 3 million adults in the United States [2]. In mainland China, the number of persons with epilepsy (PWE) from 2.30 million in 1990 increased to 9.84 million in 2015 [1]. In Arab countries, the median lifetime prevalence of epilepsy is 6.9 per 1000 and the median incidence is 89.5 per 100,000 [3]. In Europe, there are approximately 6 million PWE, with the prevalence of epilepsy at 8.23 per 1000 individuals across 53 countries [4]. Epilepsy constitutes a major determinant of the global burden of neuropsychiatric disease [5].

According to estimates, up to 50-60% of PWE experience at least one mood disorder, including depression and anxiety [6]. Anxiety disorders among PWE is approximately 22.8% and may reach 50% in specialized clinical settings [5]. The rate of depression in PWE is higher compared to patients with other chronic diseases, such as diabetes mellitus and asthma [7], as well as two to three times more common than in the general population [8].

However, there is observed variability in the prevalence of these mental disorders among PWE across the world [9]. For example, a high prevalence of depression and anxiety was reported in the United Arab Emirates (26.9% and 25.8%), China (52.6% and 33.4%), Brazil (24.4% and 39.4%), Thailand (20% and 39%), Iran (9.5% and 24.5%), USA (26.9% and 25.8%), and Canada (17.4% and 22.8%) [10].

PWE experience various difficulties that may trigger emotional burdens, such as loss of control in life due to unexpected seizures, diminished disease adaptation, and limited quality of life [10,11].

Raising awareness among health professionals regarding factors associated with anxiety and depression in this population is a challenge. Furthermore, the government and medical institutions should consider offering a multidisciplinary management program to improve psychological status among PWE [12].

The aim of this cross-sectional study was to explore factors associated with anxiety and depression among PWE.

## Materials and methods

Design, setting, and period of study

In this study, we enrolled 100 PWE visiting outpatient clinics of a public hospital in Athens, during the period from 2021 to 2022. The present study was of cross-sectional design in which the convenience sampling was used.

Inclusion and exclusion criteria of the sample

Criteria for participants’ inclusion in the study were as follows: (i) age above 18 years; (ii) ability to write, read, and understand the Greek language; (iii) ability to read and sign the informed consent form; and (iv) having active epilepsy and currently taking medication. The exclusion criteria were (i) patients with a history of mental illness, (ii) patients with cognitive impairment and sight or hearing problems, and (iii) hospitalized patients.

Data collection and procedure

Collection of data was performed using the method of interview to complete the research instrument. After patients had completed their follow up, they were invited in private office to ensure privacy. The procedure lasted approximately 20 minutes.

Research instrument

The research instrument included participants’ characteristics and the Hospital Anxiety and Depression Scale (HADS) for the evaluation of anxiety and depression. Regarding participants’ demographic characteristics, we recorded the following: gender, age, marital status, educational level, profession, residency, and number of children. In addition, participants were asked about their relations with nursing, medical staff, and other PWE, whether they kept a medical note describing the illness and reported the problem in case of emergency, whether they avoided situations in which a crisis would be dangerous, whether they smoked or drank, and if they went out at night.

Assessment of anxiety/depression

Participants’ mental health (depression and anxiety) was measured by the HADS, which was proposed by Zigmond and Snaith [13]. The scale consists of 14 questions that assess how patients felt during the previous week. Patients are able to answer every question on a four-point Likert scale from 0 to 3. Seven of the 14 questions measure depression and the other seven measure anxiety. The scores attributed to questions are summed up separately for anxiety and depression, leading to two scores ranging between 0 and 21. Higher scores indicate increased levels of anxiety or depression [14]. In addition, for both scores, the following cut-off value has been proposed and widely used in the literature: a score of 0-8 does not indicate anxiety or depression, and a score > 8 indicates anxiety/depression [15]. The HADS has been tested for its validity and reliability in the Greek population [14,15].

Ethical considerations

The present study was approved by the research committee of the hospital. Participants who met the entry criteria were informed by the researcher about the purposes of this research. Data collection guaranteed anonymity and confidentiality. All subjects had been informed of their rights to refuse or discontinue participation in the study, according to the ethical standards of the Declaration of Helsinki (1989) of the World Medical Association.

Statistical analysis

Categorical data are presented with absolute and relative (%) frequencies, while continuous ones with mean, standard deviation, median, and interquartile range, according to the normality of the data (checked with Kolmogorov-Smirnov normality test and graphically with histograms). The X2 test of independence (with Fisher’s exact adjustment wherever applicable) was used to assess the association between anxiety/depression levels and patient characteristics, as well as the non-parametric Mann-Whitney test. In addition, multiple logistic regression was performed to assess the impact of patient’s characteristics (independent factors) on their anxiety/depression. Results are presented as odds ratio (OR) and 95% confidence interval (95% CI). The observed significance level of 5% was considered statistically significant. All statistical analyses were performed using SPSS version 25 statistical program (IBM Corp., Armonk, NY).

## Results

Sample description

Table 1 shows the demographic characteristics of the sample. In particular, 35% of the sample were men, 62% were under 50 years old, 40% were single, 46% had a university education, 55% were employees, 83% were living in Attica, and 58% did not have children.

**Table 1:** Distribution of the sample according to demographic characteristics (n = 100)

Characteristics	n (%)
Gender	
Male	35 (35.0%)
Female	65 (65.0%)
Age	
18-30	26 (26.0%)
31-40	36 (36.0%)
41-50	15 (15.0%)
51-60	13 (13.0%)
61-70	8 (8.0%)
>70	2 (2.0%)
Status	
Single	40 (40.0%)
Married	31 (31.0%)
Divorced	9 (9.0%)
Living together	12 (12.0%)
Widowed	8 (8.0%)
Education level	
Primary school	10 (10.0%)
High school	33 (33.0%)
University	46 (46.0%)
MSc/PhD	11 (11.0%)
Job	
Unemployed - household	25 (25.0%)
Students	13 (13.0%)
Civil servant	12 (12.0%)
Private employee	30 (30.0%)
Freelancer	13 (13.0%)
Pensioner	7 (7.0%)
Residency	
Attica	83 (83.0%)
County	17 (17.0%)
Number of children	
0	58 (58.0%)
1	26 (26.0%)
2	14 (14.0%)
>2	2 (2.0%)

Table 2 presents the distribution of other characteristics. More specifically, the majority of patients had very good relations with nursing and medical staff (76% and 78%, respectively) and 27% maintained relations with other PWE. In addition, 50% avoided situations in which a crisis could be dangerous, 29% were keeping a medical note describing their illness, 77% were smokers, 25% were drinking alcohol, 48% were driving, and 28% went out at night.

**Table 2:** Distribution of the sample according to other characteristics (n = 100)

Characteristics	n (%)
Relations with the nursing staff	
Very good	76 (76.0%)
Good	20 (20.0%)
Moderate	4 (4.0%)
Relations with the medical staff	
Very good	78 (78.0%)
Good	17 (17.0%)
Moderate	5 (5.0%)
Relations with other persons with epilepsy (PWE)	
Yes	27 (27.0%)
No	73 (73.0%)
Do you keep a medical note describing the illness and reporting the problem in case of emergency?	
Yes	29 (29.0%)
No	55 (55.0%)
Sometimes	16 (16.0%)
Do you avoid situations in which a crisis would be dangerous?	
Yes	60 (60.0%)
No	40 (40.0%)
Do you smoke?	
Yes	77 (77.0%)
No	23 (23.0%)
Do you consume alcohol?	
Yes	25 (25.0%)
No	39 (39.0%)
Sometimes	36 (36.0%)
Do you drive?	
Yes	48 (48.0%)
No	52 (52.0%)
Do you go out at night?	
Yes	28 (28.0%)
No	33 (33.0%)
Sometimes	39 (39.0%)

Anxiety/depression

Table 3 presents results regarding anxiety/depression. A total of 58% and 48% had a HADS score higher than 8, meaning they experienced anxiety and depression, respectively. Median scores were 9 and 8 for anxiety and depression, respectively.

**Table 3:** Anxiety and depression (n = 100) HADS: Hospital Anxiety and Depression Scale.

	n (%)	Mean (SD)	Median (IQR)
Anxiety		9.6 (4.5)	9 (7-13)
No (HADS score 0-8)	42 (42.0%)		
Yes (HADS score >8)	58 (58.0%)		
Depression		8.7 (4.7)	8 (6-12)
No (HADS score 0-8)	52 (52.0%)		
Yes (HADS score >8)	48 (48.0%)		

Factors associated with anxiety/depression

Tables 4, 5 present associated factors with anxiety/depression. A statistically significant association was observed between anxiety and age (p = 0.002) (Table 4). More specifically, participants over 40 had a higher rate of anxiety (65.8%) than younger patients (53.2%). There was no statistically significant association with other variables. A statistically significant association was observed between depression and gender (p = 0.044), age (p = 0.001), marital status (p = 0.036), educational level (p = 0.003), job (p = 0.025), residency (p = 0.041), and whether they went out at night (p = 0.009) (Table 5). More specifically, female participants had a higher rate of depression (55.4%) than men (34.3%). Participants over the age of 40 had a higher rate of depression (68.4%) than younger patients (35.5%), as well as the divorced/widowed patients had a higher rate of depression (76.5%) than single and married ones (42.5% and 41.9%, respectively). Participants with primary education had a higher rate of depression (90%) than those with secondary and university education (57.6% and 35.1%, respectively). Unemployed had a higher rate of depression (68%) than pensioners and employees (55% and 36.4%, respectively). Participants living in a country area had a higher rate of depression (70.6%) than those living in Attica (43.4%). Similarly, those who did not go out at night had higher rates of depression (66.7%).

**Table 4:** Factors associated with anxiety

	Anxiety		
	No, n (%)	Yes, n (%)	p-value
Gender			0.329
Male	17 (48.6%)	18 (51.4%)	
Female	25 (38.5%)	40 (61.5%)	
Age			0.002
≤40	29 (46.8%)	33 (53.2%)	
>40	13 (34.2%)	25 (65.8%)	
Status			0.080
Single	15 (37.5%)	25 (62.5%)	
Married	23 (53.5%)	20 (46.5%)	
Divorced/widowed	4 (23.5%)	13 (76.5%)	
Education level			0.247
Primary school	3 (30.0%)	7 (70.0%)	
High school	11 (33.3%)	22 (66.7%)	
University/MSc-PhD	28 (49.1%)	29 (50.9%)	
Job			0.258
Unemployed/household	7 (28.0%)	18 (72.0%)	
Students/pensioners	9 (45.0%)	11 (55.0%)	
Employees	26 (47.3%)	29 (52.7%)	
Residency			0.940
Attica	35 (42.2%)	48 (57.8%)	
County	7 (41.2%)	10 (58.8%)	
Children			0.501
No	26 (44.8%)	32 (55.2%)	
Yes	16 (38.1%)	26 (61.9%)	
Relations with the nursing staff			0.197
Very good	35 (46.1%)	41 (53.9%)	
Good	6 (30.0%)	14 (70.0%)	
Relations with the medical staff			0.648
Very good	32 (41.0%)	46 (59.0%)	
Good	8 (47.1%)	9 (52.9%)	
Relations with other persons with epilepsy (PWE)			0.286
Yes	9 (33.3%)	18 (66.7%)	
No	33 (45.2%)	40 (54.8%)	
Do you keep a medical note describing the illness and reporting the problem in case of emergency?			0.238
Yes/sometimes	9 (27.3%)	24 (72.7%)	
No	30 (50.0%)	30 (50.0%)	
Do you avoid situations in which a crisis would be dangerous?			0.224
Yes	16 (35.6%)	29 (64.4%)	
No	26 (47.3%)	29 (52.7%)	
Do you smoke?			0.108
Yes	14 (23.3%)	46 (76.7%)	
No	28 (70.0%)	12 (30.0%)	
Do you consume alcohol?			0.875
Yes/sometimes	29 (37.7%)	48 (62.3%)	
No	13 (56.5%)	10 (43.5%)	
Do you drive?			0.658
Yes	26 (42.6%)	35 (57.4%)	
No	16 (41.0%)	23 (59.0%)	
Do you go out at night?			0.096
Yes/sometimes	22 (45.8%)	26 (54.2%)	
No	19 (41.3%)	27 (58.7%)	

**Table 5:** Factors associated with depression

	Depression		
	No, n (%)	Yes, n (%)	p-value
Gender			0.044
Male	23 (65.7%)	12 (34.3%)	
Female	29 (44.6%)	36 (55.4%)	
Age			0.001
≤40	40 (64.5%)	22 (35.5%)	
>40	12 (31.6%)	26 (68.4%)	
Status			0.036
Single	23 (57.5%)	17 (42.5%)	
Married	25 (58.1%)	18 (41.9%)	
Divorced/widowed	4 (23.5%)	13 (76.5%)	
Education level			0.003
Primary school	1 (10.0%)	9 (90.0%)	
High school	14 (42.4%)	19 (57.6%)	
University/MSc-PhD	37 (64.9%)	20 (35.1%)	
Job			0.025
Unemployed/household	8 (32.0%)	17 (68.0%)	
Students/pensioners	9 (45.0%)	11 (55.0%)	
Employees	35 (63.6%)	20 (36.4%)	
Residency			0.041
Attica	47 (56.6%)	36 (43.4%)	
County	5 (29.4%)	12 (70.6%)	
Children			0.119
No	34 (58.6%)	24 (41.4%)	
Yes	18 (42.9%)	24 (57.1%)	
Relations with the nursing staff			0.489
Very good	39 (51.3%)	37 (48.7%)	
Good	12 (60.0%)	8 (40.0%)	
Relations with the medical staff			0.315
Very good	40 (51.3%)	38 (48.7%)	
Good	11 (64.7%)	6 (35.3%)	
Relations with other persons with epilepsy (PWE)			0.069
Yes	10 (37.0%)	17 (63.0%)	
No	42 (57.5%)	31 (42.5%)	
Do you keep a medical note describing the illness and reporting the problem in case of emergency?			0.573
Yes/sometimes	22 (48.9%)	23 (51.1%)	
No	30 (54.5%)	25 (45.5%)	
Do you avoid situations in which a crisis would be dangerous?			0.423
Yes	24 (48.0%)	26 (52.0%)	
No	28 (56.0%)	22 (44.0%)	
Do you smoke?			0.621
Yes	39 (50.6%)	38 (49.4%)	
No	13 (56.5%)	10 (43.5%)	
Do you consume alcohol?			0.178
Yes/sometimes	35 (57.4%)	26 (42.6%)	
No	17 (43.6%)	22 (56.4%)	
Do you drive?			0.826
Yes	25 (52.1%)	23 (47.9%)	
No	25 (54.3%)	21 (45.7%)	
Do you go out at night?			0.009
Yes/sometimes	41 (61.2%)	26 (38.8%)	
No	11 (33.3%)	22 (66.7%)	

Impact of participants’ characteristics on their anxiety/depression

Multiple logistic regression was performed to estimate the effect of potential confounders (characteristics) on participants' anxiety and depression. Table 6 shows that female patients were 10 times more likely to develop depression than men (OR = 10.49 (95% CI: 1.31-84.14), p = 0.027).

**Table 6:** Impact of participants’ characteristics on anxiety and depression

Parameters		OR(95%CI)	p-value
Anxiety	Age		
	≤40	Ref. Cat.	
	>40	0.55 (0.15-1.97)	0.358
Depression	Gender		
	Male	Ref. Cat.	
	Female	10.49 (1.31-84.14)	0.027
	Age		
	≤40	Ref. Cat.	
	>40	0.73 (0.04-14.17)	0.834
	Marital status		
	Single	Ref. Cat.	
	Married	4.65 (0.38-56.49)	0.228
	Divorced/widowed	34.01 (0.47-2438.26)	0.106
	Education level		
	Primary school	Ref. Cat.	
	High school	1.84 (0.02-165.55)	0.791
	University/MSc-PhD	4.75 (0.03-783.45)	0.55
	Job		
	Unemployed/household	Ref. Cat.	
	Students/pensioners	13.21 (0.63-275.14)	0.096
	Employees	0.94 (0.10-8.75)	0.959
	Residency		
	Attica	Ref. Cat.	
	County	0.72 (0.07-7.91)	0.79
	Do you go out at night?		
	Yes/sometimes	Ref. Cat.	
	No	0.40 (0.03-4.99)	0.474

## Discussion

The results showed that PWE over the age of 40 years experienced anxiety and depression. A possible interpretation is that individuals of this age perceiving they are unable to perform prior functional roles or activities experience anxiety and depression. According to Acharya et al. [16], an additional reason is underdiagnosis in this age group and the absence of specialized physicians in the diagnostic process. Contrariwise, Escoffery et al. [17] argue that treatment is less effective in PWE under 30 years of age compared to older ones, which is possibly attributed to deficits in self-management and necessary skills.

Also, results revealed depression in women. Indeed, epilepsy hinders women's ability to care for family, imposes role changes, frequent hospitalizations, and financial burdens that trigger or reinforce depression. Hajszan et al. [18] stated that depression in women with epilepsy may reflect a state of hormonal deficiency and disorders in reproductive function. According to Gopinath et al. [19], comorbidities, lower employment rates, and higher anxiety occur more frequently in females. Compared to men, women face more difficulties in finding life partners and an increased risk of divorce, even when the clinical profiles of epilepsy are similar [19].

In the present study, divorced PWE experienced depression, which is explained by the lack of companionship and communication with their partner. Indeed, lack of support has negative consequences for physical and mental health [20-22]. Tedrus et al. [23] demonstrated that psychiatric comorbidity and epilepsy duration were positively associated with divorce. Marriage is less common in PWE compared to patients with other chronic diseases or the general population [23]. A study by Motamedi et al. [24] showed that 39% of PWE living alone perceived epilepsy as an obstacle to marrying the ideal partner (n = 179). On the other side of the coin, caregiver depression is a significant contributor to depression in PWE [25].

Regarding educational background, PWE with primary education experienced depression. Possibly, they are less likely to access healthcare services, face difficulties in understanding the provided information by health professionals, fail to communicate effectively with them, and confront barriers in learning self-care skills. Additionally, PWE with a low educational level may have poor coping strategies, which in turn lead to social isolation and reduced psychosocial adjustment. PWE who are unable to read and write are over four times more likely to develop depression compared to those of higher school graduates. Having lower educational status, early onset of illness, poor social support, high perceived stress, high seizure frequency, and polytherapy were factors associated with depression [8].

As for employment status, the unemployed PWE experienced depression. Interestingly, there are strong associations between epilepsy and low education and income due to difficulties in completing school or experiencing seizures and the side effects of medication. All these parameters subsequently consist of an obstacle to independent living [2]. However, many factors are held to be affecting the employability of PWE, such as clinical (uncontrolled seizure), psychological (perceived social stigma), socioeconomic (low education level), and political (driving restriction) factors [26].

PWE living in a country had a higher rate of depression than those living in Attica. It is widely accepted that the area of residence and work either protects health or creates risks. Factors more common in rural society such as economic stagnation, poverty, and low insurance rates undermine the provision of health care.

Although the importance of screening and treating anxiety and depression in PWE is increasingly recognized both in contemporary literature and guideline recommendations, in clinical practice, they remain underdiagnosed and undertreated [9,10]. It is not rare that health professionals often ignore, overlook, or are unable to recognize mental burden, they tend to focus on the biological dimension of the disease and act only when the patient complains. Nurses are called to screen and understand the comorbidity of anxiety/depression and confront possible obstacles, such as lack of proper training, skills, and available time [27,28].

Limitations of the study

In this cross-sectional study, there was no evidence of a causal relationship between anxiety/depression and characteristics of patients. The method of convenience sampling in a single-center study in Attica is not representative of all PWE living in Greece, thus limiting the generalizability of the results. Moreover, there was no next measurement in time that would allow the evaluation of possible changes in all dimensions under assessment. Although many significant associations were observed, the sample size might be a small one. In addition, the presence of anxiety and depression was only explored through questionnaires and not by psychiatric evaluation.

The HADS is a widely used research tool that permits comparison with other research studies on a global scale.

## Conclusions

According to the present results, an association was observed between depression and gender, age, marital status, educational level, job, residency, and whether they went out at night. In terms of anxiety, results revealed an association between anxiety and age. Interestingly, in-depth knowledge of factors associated with the frequently encountered dyad of anxiety/depression and epilepsy is supposed to help guide clinical decision-making. Nurses as health professionals should adopt an approach model based on scientific information, guidance, and patient support combined with evidence-based knowledge as well as to strengthen the expression of questions regarding the disease and its treatment. Moreover, they need to ensure that PWE receive comprehensive care, including access to health promotion resources and counseling for risk behaviors.
